# Mitochondrial Dysfunction: The Nexus of Aging, Dyslipidemia, and CKD

**DOI:** 10.1016/j.ekir.2025.01.010

**Published:** 2025-01-10

**Authors:** Yu-Hsiang Lin, Kuo-Jen Lin, Chih-Hsiang Chang

**Affiliations:** 1 Department of Urology, Chang Gung Memorial Hospital-Linkou, Taoyuan City, Taiwan; 2 School of Medicine, Chang Gung University, Taoyuan City, Taiwan; 3 Department of Nephrology, Chang Gung Memorial Hospital-Linkou, Taoyuan City, Taiwan

To the Editor:

We read with great interest the recent article by Huang *et al.*, which provides compelling insights into the mechanisms underlying vascular calcification. Their discussion highlights mitochondrial dysfunction as a key contributor to vascular calcification, particularly in the context of chronic kidney disease.^1^ However, we were intrigued by the complexity of the interplay between mitochondrial dysfunction and dyslipidemia in the etiology of vascular calcification, as discussed in other recent works.^2^

This dual perspective—mitochondrial dysfunction versus dyslipidemia as the primary driver of vascular calcification—raises an important question: could mitochondrial dysfunction serve as the unifying mechanism underlying both dyslipidemia and vascular calcification? Huang *et al.*'s findings support this hypothesis, because mitochondrial dysfunction not only promotes vascular calcification but is also closely associated with dyslipidemia, as described in related studies.^3^

To further illustrate this concept, we have prepared a schematic representation (Figure 1) that outlines the central role of mitochondrial dysfunction in connecting aging-associated hormonal deficiency, vascular calcification, dyslipidemia, and chronic kidney disease.

**Figure 1:**
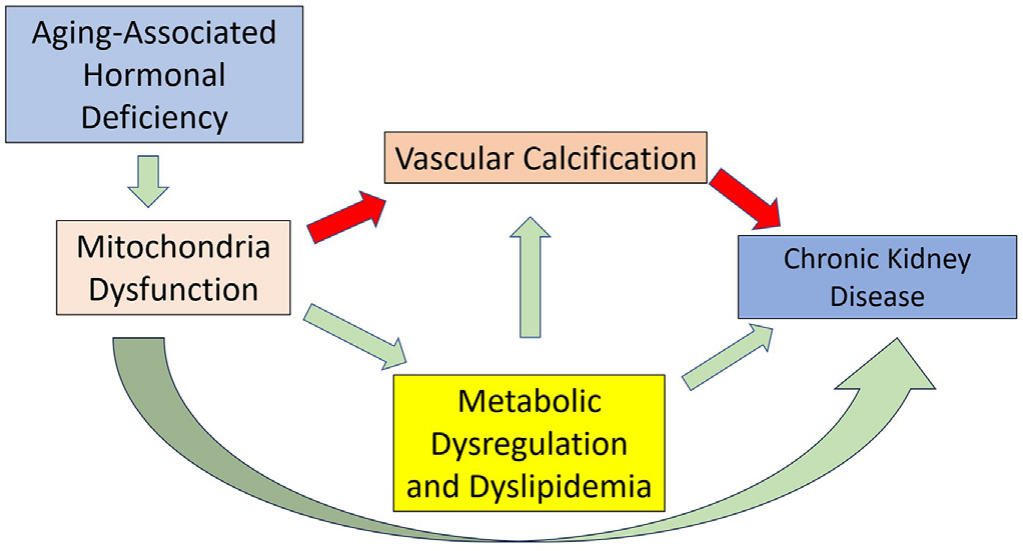
The role of mitochondrial dysfunction in vascular calcification, dyslipidemia, and CKD. This figure illustrates the central role of mitochondrial dysfunction in connecting aging-associated hormonal deficiency, vascular calcification, dyslipidemia, and CKD. Red arrows represent established relationships supported by the current literature, such as the contribution of mitochondrial dysfunction to vascular calcification and CKD. Green arrows indicate the proposed mechanisms based on emerging insights, including the role of mitochondrial dysfunction in metabolic dysregulation and its impact on dyslipidemia and CKD. CKD, chronic kidney disease.

In the aging male population, the pathophysiology becomes even more intricate. Age-related hormonal changes, including benign prostatic hyperplasia–induced nocturia disrupt circadian rhythms and subsequently impair antidiuretic hormone secretion. This disruption exacerbates nocturnal polyuria, creating a vicious cycle of nocturia and circadian rhythm disturbance.^4^ Moreover, these alterations in the circadian homeostasis negatively impact the hypothalamic-pituitary-gonadal axis, resulting in decreased testosterone levels. As demonstrated in previous studies, testosterone deficiency exacerbates mitochondrial dysfunction, thereby linking hormonal deficiency to vascular calcification and dyslipidemia.^5^

Similarly, postmenopausal women face a distinct yet convergent challenge. The loss of estrogen—a critical regulator of mitochondrial function—further predisposes this population to mitochondrial dysfunction and its downstream effects, including dyslipidemia and vascular calcification.S1 Mitochondrial dysfunction has also been implicated in the progression of chronic kidney disease, as highlighted in recent reviews.S2

In conclusion, we propose that mitochondrial dysfunction represents a central axis connecting hormonal deficiency, dyslipidemia, vascular calcification, and chronic kidney disease. This perspective underscores the need for further research into mitochondrial-targeted therapies that could simultaneously address these interconnected pathologies. We commend Huang *et al.* for their important contributions and hope this letter stimulates additional dialogue on this multifaceted topic.

## Disclosure

All the authors declared no competing interests.

### Declaration of AI and AI-Assisted Technologies in the Writing Process

During the preparation of this work, the authors used ChatGPT in order to improve readability and language. After using this tool, the authors reviewed and edited the content as needed and take full responsibility for the content of the publication.

## Supplementary Material

Supplementary File (PDF) **Supplementary References**.

## Footnotes

Supplementary File (PDF)

**Supplementary References.**

## References

[1] Huang J., Hao J., Wang P., Xu Y. (2024). The role of mitochondrial dysfunction in CKD-related vascular calcification: From mechanisms to therapeutics. Kidney Int Rep.

[2] Demer L.L., Tintut Y., Parhami F. (2002). Novel mechanisms in accelerated vascular calcification in renal disease patients. Curr Opin Nephrol Hypertens.

[3] Zong Y., Li H., Liao P. (2024). Mitochondrial dysfunction: Mechanisms and advances in therapy. Signal Transduct Target Ther.

[4] Lin Y.H., Wu C.T., Juang H.H. (2024). Exploring the complex interplay: BPH, nocturia, and aging male health. World J Urol.

[5] Rovira-Llopis S., Bañuls C., de Marañon A.M. (2017). Low testosterone levels are related to oxidative stress, mitochondrial dysfunction and altered subclinical atherosclerotic markers in type 2 diabetic male patients. Free Radic Biol Med.

