# Supplementary material for: Mitochondrial Dysfunction: The Nexus of Aging, Dyslipidemia, and CKD
Source: Kidney Int Rep. 2025 Jan 10;10(3):973–4. doi: 10.1016/j.ekir.2025.01.010 (PMC11993211; doi:10.1016/j.ekir.2025.01.010)
Supplement: Supplementary File (PDF) — Supplementary References. [file mmc1.pdf]

S1. Klinge CM. Estrogenic control of mitochondrial function. *Redox Biol*. Apr 2020;31:101435. doi:10.1016/j.redox.2020.101435
S2. Zhang X, Agborbesong E, Li X. The Role of Mitochondria in Acute Kidney Injury and Chronic Kidney Disease and Its Therapeutic Potential. *Int J Mol Sci*. Oct 19 2021;22(20)doi:10.3390/ijms222011253
